# Supplementary material for: Using attribution theory to explore the reasons adults with hearing loss do not use their hearing aids
Source: PLoS One. 2020 Sep 4;15(9):e0238468. doi: 10.1371/journal.pone.0238468 (PMC7473559; doi:10.1371/journal.pone.0238468)
Supplement: S1 Appendix — (DOCX) [file pone.0238468.s001.docx]

**S1 Appendix: Interviewer’s Narrative Interview Script**

As you know, we’re recording stories about people’s hearing loss and their decision-making process to decline hearing aids. Would you mind if I audio recorded this interview and took brief notes? Ok, I’m going to turn on the recorder now and confirm your permission to record.

***TURN ON THE RECORDER***

I am conducting this interview as a part of a research study aimed at better understanding people with hearing loss who choose not to use hearing aids. Would you mind if I audio recorded this interview and took brief notes?

Before our meeting, I sent you the link to a questionnaire. I see that you consented to participate in this study – is this correct? Do you have any questions before we begin?

Remember that you do not have to share anything or answer any question(s) that you are not comfortable with. You can stop your story at any time if you so choose. Once we are done, this recording will be transcribed, and your information will not be attached to the recording or the transcript in any way. Your name will not appear in the data, nor will the names of anyone you might mention. Pseudonyms will be used in any papers written for any publications or presentations. Are you ready to begin?

Today, I’m going to ask you to tell me a story about your experience learning about your hearing loss and your decision-making process to decline hearing aids. During your story, I will remain quiet until you reach the end. After your story, I will ask you some follow-up questions.

There’s no right way to tell your story but I am interested in hearing about it from the beginning and then step-by-step until the end…Like any novelist, you can edit the chapters of your story you already wrote—you can add more detail or retell certain sections, whenever and however you would like. I’m also interested in the cast of characters. In other words, who else was present during hearing loss diagnosis and your decision-making process or who else have you talked to about your hearing loss? And so forth…

Do you have any questions?

Ok, now it’s time for you to tell me your story. Why don’t you start by telling me about when you were diagnosed with hearing loss and when it was recommended that you get fit with hearing aids. Then please share with me how your decision-making process unfolded and when you made the choice NOT to follow through with hearing aids?
