# Supplementary material for: Using attribution theory to explore the reasons adults with hearing loss do not use their hearing aids
Source: PLoS One. 2020 Sep 4;15(9):e0238468. doi: 10.1371/journal.pone.0238468 (PMC7473559; doi:10.1371/journal.pone.0238468)
Supplement: Attachment — Submitted filename: Response to Reviewers.docx [file pone.0238468.s002.docx]

**“Using attribution theory to explore the reasons adults with hearing loss do not use**

**their hearing aids”**

**(PONE-D-20-12039), submitted to**

***PLOS One***

Below you will find our responses to each reviewer’s comments. We were able to address all of the reviewers’ comments. We have also added an Appendix that includes our interview protocol.

***Response to Reviewer 1’s feedback***

Thank you, Dr. Diviani, for your helpful feedback.

1) ***I find it strange to see that one of the participants reported using a HA during all waking hours. Why was he not excluded? Even if he identified as a non-user, I think his perspective is different from the others.*** We understand and appreciate this perspective from both Reviewers 1 and 2 (see Reviewer 2’s comment (5)). We did not exclude this participant (or any others) based on their reported hearing aid (HA) use times because we are interested in the participants’ *perceptions* of themselves (and the stories they tell themselves and others) because research shows that human behaviors are shaped by our perceptions (for more detail see Moen, 2006, p. 63). Sometimes humans’ perceptions align with measurable quantitative data, sometimes they do not. For example, I could be a “healthy” weight according to the World Health Organization and measurements of Body Mass Index, but if I perceive I am “fat” I might change my eating behaviors and go on a diet. Or I could be 45 kilos overweight (“obese” according to the World Health Organization and measurements of Body Mass Index) but perceive that I am “healthy” and not engage in any behaviors that affect my weight.

Thus, in the case of our study and current MS, if a participant thought of themselves as a HA non-user, then they are behaving (and making meaning in their life) accordingly. Their perception of who they are is what ultimately matters and their perception as a HA non-user actually *matches* the other participants’. We agree that it would be interesting to know how quantitative information, such as data logging, correlates with other qualitative variables (e.g., self-identity), however it is not standard procedure to include quantitative details about the sample in a qualitative narrative study such as ours. For example, reporting frequencies or other quantitative data erases the nuance of analysis (reducing it to a question of “how much” as opposed to a question of quality). We have revised our MS and clarified for the reader our decision-making process when it came to including all people who identify as “non-users” (p. 9 footnote).

2) ***Details about software and tools (e.g., brand names) are not needed.*** Thank you for your feedback. We eliminated these details throughout our “Methods” sections.
3) ***Please provide a brief definition of internally vs externally motivated reasons in the first paragraph.*** We included a definition on p. 3.
4) ***Table 2: I find the themes + description of the themes to be a bit redundant.*** We agree with Reviewer 1 that many of the themes’ descriptions are redundant with their theme titles. However, for some remaining themes the descriptions add much-needed clarification that the themes’ labels cannot convey if they stand alone (e.g., stigmatization theme). Thus, we have decided to keep both the themes and descriptions in the Table for consistency/parallelism across the Table.

***Response to Reviewer 2’s feedback***

We are grateful for Reviewer 2’s comments. As they commented, “The authors realize these limitations. Almost all of them are listed in the Limitations and Future Directions section.” We have further addressed them below and expanded on them in the MS when possible (of particular note is Reviewer 2’s 5th question).

1) ***Marginally small sample (n = 20) for the narrative study design.*** When saturation is reached (i. e. no new identities emerge from the stories) no additional data points are required for analysis (Fusch & Ness, 2015). As noted in the “Verification procedures” section, saturation for our study was reached at Interview #11 (as determined retrospectively). We made an *a priori* decision to collect stories from 20 individuals based on the saturation points of other thematic narrative analyses conducted with samples of low-incidence clinical populations (e.g., Flood-Grady & Koenig Kellas, 2019). We also selected and N = 20 to give us enough stories to reasonably engage in referential adequacy.
2) ***Too big age range (27 to 91 years). Too big PTA range (15 to 50 dB, i.e. from normal hearing to moderate HL).*** Our study’s aim was to gather narratives from adults who did not use their prescribed HAs. Our final sample includes individuals all over the age 18-years-old, thus they fit the operational definition of adulthood. Furthermore, all of these individuals were diagnosed with permanent hearing loss and prescribed HAs by a hearing healthcare provider—our operational definition for hearing loss was not rooted in a particular degree or shape of hearing loss. Nonetheless, we recognize the experimental control that could be afforded by a study with a different design (and a more constrained participant eligibility criteria) than the one we used for the current study.
3) ***Non-necessity COULD be a good non-usage reason for a person with close to normal (or even normal) hearing.*** Reviewer 2 is correct—in fact, all of the attributions that were illuminated in our study could be considered “good” reasons for HA nonuse. However, the aim of our study was to bring to light what reason HA non-users perceive as barriers to HA use. It was not our goal to assign value (or validity) to the reasons.
4) ***Only white people are included. Any reasons for it?*** Reviewer 2 is again correct. As we mentioned and discussed in our initial MS submission, our sample primarily consisted of White individuals, despite our public recruiting via social media, our lab’s website, etc. This was not intentional nor is it ideal, as we mention in the “Limitations and Future Directions” section of our paper. We elaborated more on why this can be a problem and how to potentially solve it in the future (p. 27).
5) ***How could a person wearing HAs “during most waking hours” or even “during all waking hours” identify her(him)self as “non-user”? Can we trust other narrative information from such study participant? (3 persons with above-mentioned wearing time and 1 person wearing HAs during about a half of waking hours, which totals to 1/5 of study sample).*** We understand and appreciate this perspective from both Reviewers 1 and 2 (see our response to Reviewer 1’s comment (1) above for details). We did not exclude any participants based on their reported HA-use times because we are interested in the participants’ *perceptions* of themselves, not a quantitative measurement/assessment of whether or not they should be categorized as a non-user.
